# Clinical Effectiveness and Safety of Oral Semaglutide in a Real-World Cohort of Patients with Heart Failure with Reduced Ejection Fraction, Type 2 Diabetes and Obesity: A Propensity Score-Matched Analysis

**DOI:** 10.3390/ph19060894

**Published:** 2026-06-05

**Authors:** Alicia Trenas-Calero, Nuria Prieto-Laín, Miguel A. Pérez-Velasco, Claudia Padilla-Leiva, José M. Pérez-Ruiz, Fátima I. Ruíz-Rodríguez, Ricardo Gómez-Huelgas, María-Rosa Bernal-López, Luis M. Pérez-Belmonte

**Affiliations:** 1 IBIMA Plataforma BIONAND, Instituto de Investigación Biomédica de Málaga, 29590 Málaga, Spain; aliciatrenascalero@gmail.com (A.T.-C.); prietolain@hotmail.com (N.P.-L.); miguelpv89@gmail.com (M.A.P.-V.); claudiapadillaleiva@gmail.com (C.P.-L.);; 2 Servicio de Medicina Interna, Hospital Regional Universitario de Málaga, 29010 Málaga, Spain; 3 Servicio de Cardiología, Hospital Regional Universitario de Málaga, 29010 Málaga, Spain; 4 Centro de Investigación Biomédica en Red Fisiopatología de la Obesidad y Nutrición (CIBERobn), Instituto de Salud Carlos III, 28029 Madrid, Spain; 5 Departamento de Medicina y Dermatología, Facultad de Medicina, Universidad de Málaga (UMA), 29010 Málaga, Spain; 6 Centro de Investigación Biomédica en Red Enfermedades Cardiovasculares (CIBERCV), Instituto de Salud Carlos III, 28029 Madrid, Spain

**Keywords:** semaglutide, heart failure, reduced left ventricular ejection fraction, type 2 diabetes, obesity

## Abstract

**Bacground/Objectives**: There is limited evidence on the role of glucagon-like peptide-1 receptor agonists in heart failure. We aimed to analyze the clinical efficacy of oral semaglutide in terms of health status and change in body weight in patients with heart failure with reduced ejection fraction, type 2 diabetes, and obesity. **Methods**: This observational, retrospective, real-world study included patients treated with oral semaglutide (Oral-Sema Group) and without glucagon-like peptide-1 receptor agonists (Control Group). The primary outcome was heart failure status, defined as a ≥5 point difference in the Kansas City Cardiomyopathy Questionnaire total symptom score, and change in body weight at 24 months. **Results**: After 1:1 propensity score matching, 162 patients were included in each group (mean age 71.0 years, mean body mass index 32.1, 52.9% females). Patients in the Oral-Sema Group were more likely to have improvement in heart failure health status from baseline to 24 months (OR: 2.45; 95%CI: 1.25–3.65; *p* = 0.012). The mean change in body weight was −8.0 ± 2.1 kg in patients with oral semaglutide and −1.9 ± 1.0 kg in control patients (*p* < 0.01). After treatment, there were negative correlations between the Kansas City Cardiomyopathy Questionnaire total symptom score and body weight (r = −0.558, *p* < 0.01) and glycated hemoglobin (r = −0.491, *p* = 0.017). It had good tolerability and safety. **Conclusions**: Oral semaglutide was associated with an improvement in heart failure health status and weight loss in patients with heart failure with reduced ejection fraction, type 2 diabetes, and obesity. Further research on glucagon-like peptide-1 receptor agonists in heart failure with reduced ejection fraction is needed.

## 1. Introduction

Despite substantial advances in pharmacological and non-pharmacological management, heart failure (HF) continues to be associated with a high burden of morbidity and mortality, with frequent hospital admissions and persistent impairment in quality of life. Outcomes are particularly unfavorable in patients who also have type 2 diabetes mellitus (DM), a common comorbidity that contributes to disease progression and adverse cardiovascular events [1,2,3].

Sodium–glucose cotransporter 2 inhibitors (SGLT-2is) have emerged as key therapeutic agents in type 2 DM because of their cardiovascular and renal protective properties. Current guidelines recommend their use in individuals with established cardiovascular disease or elevated cardiovascular risk [4,5,6]. Importantly, the benefits of dapagliflozin and empagliflozin extend to patients with heart failure with reduced ejection fraction (HFrEF), regardless of diabetes status, as demonstrated in the DAPA-HF and EMPEROR-Reduced trials [7,8]. In addition, glucagon-like peptide-1 receptor agonists (GLP-1ras) have consistently shown favorable cardiovascular and renal effects in patients with type 2 DM [9]. More recently, semaglutide has attracted increasing interest in the field of HF. A pooled analysis of the SELECT, FLOW, STEP-HFpEF, and STEP-HFpEF DM trials reported reductions in cardiovascular death and worsening HF events among patients receiving semaglutide [10]. Similarly, participants enrolled in the STEP-HFpEF and STEP-HFpEF DM studies experienced clinically meaningful improvements in symptoms and physical limitations [11,12]. Cardiovascular protection has also been demonstrated in overweight or obese individuals with established cardiovascular disease in the SELECT trial and in patients with chronic kidney disease and type 2 DM in the FLOW study [13,14]. Likewise, tirzepatide, a dual GIP (glucose-dependent insulinotropic peptide)/GLP-1ra, has shown benefits on HF-related outcomes and health status in patients with HFpEF and obesity [15]. However, evidence regarding the role of oral semaglutide in HFrEF remains limited [16].

Given the close interplay among HFrEF, obesity, and type 2 DM, together with the limited data concerning oral semaglutide in this population, we aimed to evaluate its effectiveness and safety in routine clinical practice, focusing on HF-related health status, change in body weight, and cardiovascular and renal outcomes. It is hypothesized that the use of oral semaglutide would be efficacious and safe in this group of patients.

## 2. Results

**Figure 1:**
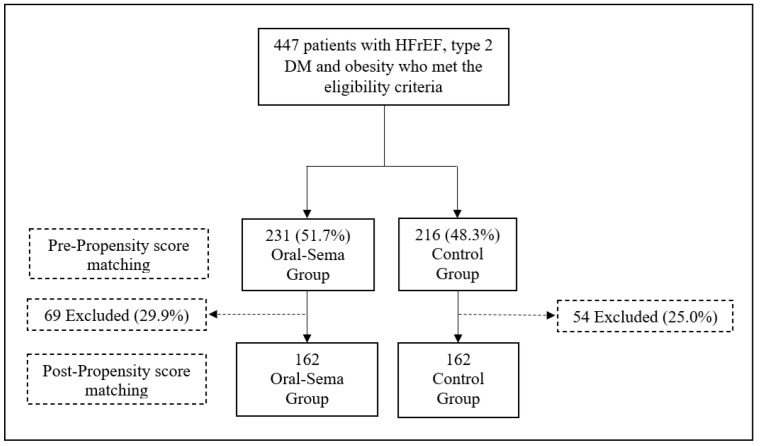
Patient flow charts for Oral-Sema versus Control group. DM: diabetes mellitus; HFrEF: heart failure with reduced ejection fraction.

A total of 447 patients with HFrEF, type 2 DM, and obesity were included in this study. Of them, 231 were included in the Oral-Sema Group and 216 in the Control Group. After PSM, 162 patients were included in each group. A total of 134/162 patients (82.7%) reached a dose of 14 mg of oral semaglutide. A flow chart for patient inclusion for both groups is shown in Figure 1. During the observation period, all patients remained under follow-up. The only discontinuations corresponded to deaths.

**Figure 2:**
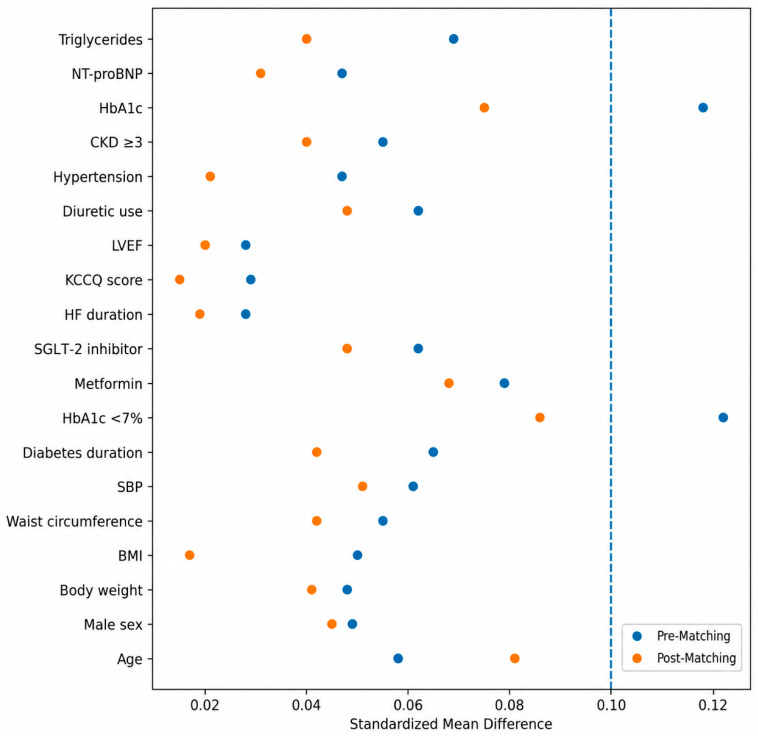
Standardized mean differences before and after propensity score matching. BMI: body mass index; CKD: chronic kidney disease; HbA1c: glycated hemoglobin; HF: heart failure; KCCQ: Kansas City Cardiomyopathy Questionnaire; LVEF: left ventricular ejection fraction; NT-proBNP: N-terminal pro-brain natriuretic peptide; SBP: systolic blood pressure; SGLT-2: sodium−glucose cotransporter 2.

Before PSM, the proportion of patients with HbA1c < 7% was lower in the Oral-Sema Group than in the Control Group (7.4% vs. 23.6%, *p* = 0.018) and the HbA1c level was higher (7.8 ± 1.3 vs. 7.0 ± 1.1%, *p* = 0.038). No other significant differences were found between the groups. After PSM, both groups were well-balanced and no differences were found. Table 1 shows the baseline sociodemographic, clinical, and treatment characteristics of patients before and after PSM. Standardized mean differences before and after PSM are shown in Figure 2.

**Figure 3:**
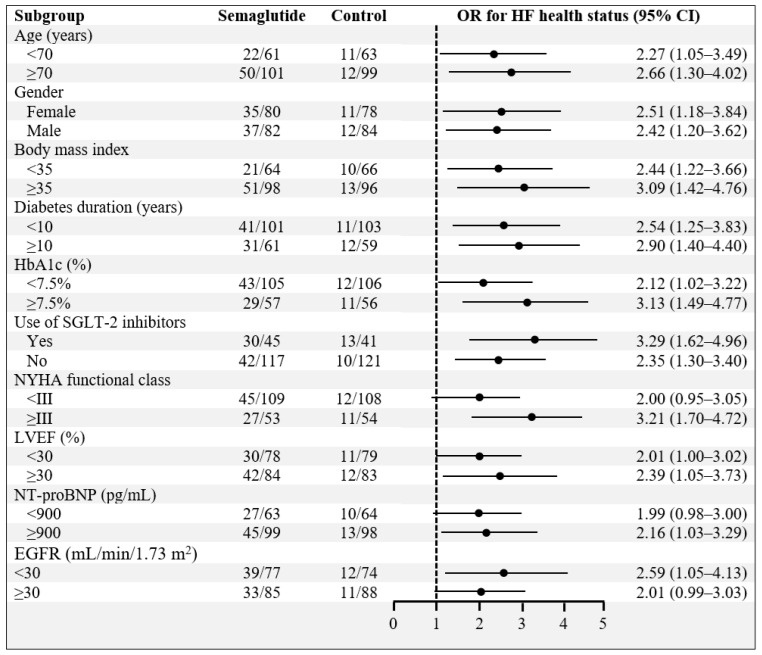
Subgroup analysis of primary outcome. Data are shown as absolute values (number of patients with events/number of patients at risk). In order to evaluate the association between treatment and study outcomes, mixed effect logistic regressions were used. The regression analysis values were expressed as odds ratio and 95% confidence interval. Values were considered to be statistically significant if *p* < 0.05. 95% CI: 95% confidence interval; EGFR: estimated glomerular filtration rate; HbA1c: glycated hemoglobin; HF: heart failure; LVEF: Left ventricular ejection fraction; NT-proBNP: N-terminal pro-brain natriuretic peptide; NYHA: New York Heart Association; SGLT-2: sodium−glucose cotransporter 2.

Patients in the Oral-Sema Group were more likely to have an improvement in HF health status from baseline to 24 months (OR: 2.45; 95% CI: 1.25–3.65; *p* = 0.012). There was an improvement of 17.5 ± 3.9 points on the KCCQ total symptom score for patients in the Oral-Sema Group compared to 6.5 ± 1.7 points for patients in the Control Group (*p* < 0.01). The mean change in body weight was −8.0 ± 2.1 kg in patients with oral semaglutide and −1.9 ± 1.0 kg in control patients (*p* < 0.01). The effects of semaglutide on HF health status appeared to be consistent across the prespecified subgroups (Figure 3).

There were also significant declines in HF events (OR: 0.84; 95% CI: 0.70–0.98; *p* = 0.018) and their individual components: emergency department visits for HF decompensation (OR: 0.86; 95% CI: 0.73–0.99; *p* = 0.024), HF hospitalizations (OR: 0.84; 95% CI: 0.69–0.99; *p* = 0.020), and unplanned outpatient visits (OR: 0.87; 95% CI: 0.75–0.99; *p* = 0.039). There were also significant reductions in cardiovascular death (OR: 0.88; 95% CI: 0.77–0.99; *p* = 0.035) and all-cause hospitalizations (OR: 0.87; 95% CI: 0.76–0.98; *p* = 0.038). No reductions were observed in all-cause death or new or worsening nephropathy. HF health status and secondary outcome measure results are shown in Table 2.

Patients treated with oral semaglutide had a greater reduction in HbA1c levels (1.0 ± 0.3 vs. 0.2 ± 0.1%, *p* < 0.01) than control patients from baseline to 24 months. There were negative correlations between the KCCQ total symptom score and body weight (r = −0.558, *p* < 0.01) and HbA1c (r = −0.491, *p* = 0.017). 

Serious adverse events were observed in 49 patients treated with once-daily oral semaglutide (30.2%). All events were gastrointestinal (30 nausea, 11 vomiting, and 8 diarrhea) and occurred during the first few weeks of treatment or after dose increases. Twenty-four patients (14.8%) had more severe symptoms and discontinued oral semaglutide. No other adverse events that could interfere with HF medication were reported during follow-up.

## 3. Discussion

In this real-world study, the use of once-daily oral semaglutide was associated with a greater improvement in HF health status in patients with HFrEF, type 2 DM, and obesity. A significant reduction in body weight was also associated with the use of oral semaglutide compared to patients who did not receive a GLP-1ra. In addition, there were reductions in HF events (including their individual components: emergency department visits for HF decompensation, HF hospitalizations, and unplanned outpatient visits), cardiovascular death, and all-cause hospitalizations. Its tolerability and safety were good, with only a few gastrointestinal adverse events.

Several pharmacological therapies have substantially improved the prognosis of patients with HFrEF, including beta-blockers, angiotensin-converting enzyme inhibitors, angiotensin receptor–neprilysin inhibitors, mineralocorticoid receptor antagonists, and SGLT-2is, all of which are currently considered pillars of guideline-directed medical therapy because of their beneficial effects on mortality and HF-related hospitalizations [17]. Among these therapies, SGLT-2is have demonstrated consistent benefits across the spectrum of HFrEF, irrespective of diabetes status, and have transformed the management of HF in recent years [7,8]. In contrast, the role of GLP-1ras in HF remains less clearly established. Although these agents have consistently demonstrated cardiovascular and renal protection in patients with type 2 DM [4,6,9], their specific effects on HF outcomes have been more heterogeneous. A large meta-analysis of cardiovascular outcome trials reported a modest but statistically significant reduction in HF hospitalizations among patients receiving GLP-1ras [9]. More recently, growing interest in the cardiometabolic effects of these agents has led to several studies evaluating their role in patients with obesity and HF.

Semaglutide has emerged as one of the most extensively studied agents in this field. A pooled analysis of the SELECT, FLOW, STEP-HFpEF, and STEP-HFpEF DM trials demonstrated reductions in cardiovascular death and worsening HF events among patients treated with semaglutide [10]. In addition, both the STEP-HFpEF and STEP-HFpEF DM trials showed clinically meaningful improvements in symptoms, physical limitations, exercise capacity, and quality of life among patients with obesity and HFpEF, regardless of DM [11,12]. The SELECT trial further demonstrated a reduction in major adverse cardiovascular events among overweight or obese individuals with established cardiovascular disease [13], while the FLOW trial reported significant renal and cardiovascular benefits in patients with type 2 DM and chronic kidney disease [14]. Similar observations have recently been reported with tirzepatide, a dual GIP/GLP-1ra, which was associated with improvements in HF outcomes and health status in patients with HFpEF and obesity [15]. Collectively, these findings support the hypothesis that therapies targeting obesity, insulin resistance, and cardiometabolic dysfunction may exert favorable effects on HF progression and related outcomes.

Despite the increasing body of evidence supporting the cardiovascular benefits of GLP-1ra, data specifically evaluating oral semaglutide in patients with HF remain limited. While its cardiovascular safety in patients with type 2 DM at high cardiovascular risk was assessed in the PIONEER-6 trial [16], its cardiovascular efficacy was evaluated in the SOUL trial [18], a placebo-controlled study including 9650 patients with type 2 DM and established atherosclerotic cardiovascular disease. In this trial, oral semaglutide significantly reduced the risk of major adverse cardiovascular events compared with placebo. Notably, 23% of the study population had HF, supporting the potential role of oral semaglutide in this clinical setting. Moreover, a recent secondary analysis of the SOUL trial [19] reported that oral semaglutide was associated with a reduction in HF events among patients with a prior history of HF. However, whereas this benefit was clearly demonstrated in patients with HFpEF, a neutral effect was observed in those with HFrEF. In contrast, our findings showed that once-daily oral semaglutide was associated with improved HF outcomes. These discrepancies between studies may be explained by several factors, including differences in primary outcome definitions, HF event adjudication, study methodology, and baseline clinical characteristics. In our cohort, patients were older, more frequently obese, had a worse NYHA functional class, and were more commonly treated with diuretics. Consistent with our findings, a recent observational study evaluating the efficacy and safety of oral semaglutide in patients with HFpEF, type 2 DM, and obesity demonstrated improvements in HF-related health status together with reductions in HF events [20]. Collectively, these findings may further support the potential role of oral semaglutide across the HF spectrum, irrespective of LVEF.

Potential mechanisms underlying the observed benefits of GLP-1 receptor agonists in HF have been proposed in previous experimental and clinical studies. These mechanisms may include favorable effects on endothelial function, inflammation, insulin resistance, natriuresis, and cardiorenal hemodynamics, as well as modulation of GLP-1 receptor signaling in cardiac tissue [21,22,23,24,25]. In addition, GLP-1 receptor agonists have been associated with improvements in glycemic control and substantial body weight reduction, which could indirectly contribute to better HF-related outcomes [21,25]. Epicardial adipose tissue has also been suggested as a potential mediator due to its role in adipokine production and myocardial energy homeostasis [26]. However, these proposed mechanisms remain speculative in the context of the present study, as no mechanistic biomarkers or pathophysiological analyses were specifically evaluated.

Obesity may also play an important role in the clinical course of HFrEF. It is a well-established risk factor for incident HF and has been associated with increased plasma volume, adverse cardiac remodeling, inflammation, fibrosis, and sodium retention, all of which may contribute to myocardial dysfunction and disease progression [27,28].

In patients with established HFrEF, obesity is associated with higher rates of HF hospitalizations and a worse burden of symptoms, but paradoxically, mild-to-moderate obesity has been linked to improved short-term survival—a phenomenon known as the “obesity paradox” [29,30]. Although this paradox may be explained by greater metabolic reserve and muscle mass, its presence has not been clearly established and obesity still increases the risk of adverse outcomes in patients with HFrEF [29,31].

Although obesity management in HFrEF remains controversial, HF guidelines recommend intentional weight reduction for patients with obesity, especially for patients with a BMI ≥ 35 kg/m^2^, to improve symptoms and cardiac function [27,30]. Weight loss strategies should prioritize lifestyle interventions and pharmacologic options can be considered, but evidence for benefits in HFrEF is less clear than in HFpEF [29,30]. This study suggests a potential beneficial association between oral semaglutide use and improved HF-related outcomes in patients with HFrEF, type 2 diabetes, and obesity. The benefits of semaglutide may be additive to those demonstrated by SGLT-2is in patients with HF, type 2 DM, and obesity, improving multiple domains of the cardiovascular-kidney-metabolic syndrome and targeting the underlying pathophysiological links among them [32].

Interestingly, beyond their clinical benefits, emerging data suggest that therapies with proven cardiovascular benefits, such as GLP-1 receptor agonists, may also reduce the environmental footprint of healthcare by decreasing HF hospitalizations and associated resource utilization [33].

For this reason, the implementation of structured treatment programs that include the use of GLP-1ras in combination with improvements in diet quality and exercise to achieve long-term weight loss and an increase in lean mass could be established as an important goal in the management of patients with type 2 DM, overweight/obesity, and HFrEF. It may even be possible to extrapolate these potential benefits to patients with overweight or obesity without type 2 DM [31,34,35,36].

This study is noteworthy in that it provides the first real-world evidence on the impact of once-daily oral semaglutide on HF-related outcomes in patients with HFrEF, type 2 DM, and obesity. Nevertheless, the results should be interpreted in light of several limitations. First, despite the use of PSM and mixed-effects logistic regression models to adjust for potential confounders, the observational design precludes the complete exclusion of residual or unmeasured confounding factors. Moreover, information on certain relevant clinical variables, such as baseline levels of physical activity or quality of life, was not available. Treatment allocation was determined by clinicians’ judgment in routine clinical practice rather than by randomization, which may have introduced selection bias and confounding by indication despite the use of propensity score matching and multivariable adjustment strategies. Second, although the incidence of primary outcome events was sufficient for analysis, the relatively small number of secondary outcome events limits the robustness of conclusions regarding the effects of semaglutide on these endpoints. In addition, the assessment of health status was restricted to the KCCQ total symptom score, without separate analyses of the individual domains. Additionally, the subgroup analyses were exploratory; therefore, their results should be interpreted with caution. Third, background HF therapy could be modified at the discretion of treating clinicians during follow-up, making it difficult to attribute the effects observed exclusively to starting oral semaglutide. A substantial proportion of patients were concomitantly receiving SGLT-2is, whose known benefits may have influenced the results. Furthermore, lifestyle interventions, including dietary counseling and physical activity, together with diuretic dose adjustments according to functional status, may have influenced the observed changes in cardiovascular outcomes and body weight. As no specific monitoring strategies or recommendations aimed at preserving muscle mass were implemented, the possibility of semaglutide-associated muscle mass loss in a proportion of patients cannot be excluded. In addition, treatment adherence and characterization of adverse events were not formally assessed, which may have further influenced the findings. Fourth, the follow-up duration was limited to 24 months, which was selected because it allowed the inclusion of a representative number of patients treated with oral semaglutide since its commercialization in Spain while enabling the assessment of mid-term clinical outcomes. Therefore, the long-term effects of oral semaglutide in patients with HFrEF remain to be established. Further studies with longer follow-up periods are warranted to better evaluate the durability of clinical benefits and long-term safety outcomes. Fifth, given the real-world observational nature of the study, it was not possible to directly explore the biological mechanisms underlying the observed effects of oral semaglutide. Additional studies focusing on cardiovascular and metabolic pathways, including the role of GLP-1ra–associated weight loss, are warranted in order to better define their efficacy and safety in patients with HF and obesity. Finally, the authors acknowledge that the sample size of this prospective real-world cohort was relatively limited. All eligible patients with HFrEF, type 2 DM, and obesity who completed follow-up in the heart failure units were included. Larger multicenter studies with extended follow-up durations would be required to confirm and further substantiate these findings.

## 4. Materials and Methods

### 4.1. Study Design and Patients

This observational, retrospective, real-world study was performed on patients with HFrEF, type 2 DM, and obesity treated with once-daily oral semaglutide (Oral-Sema Group) or without semaglutide or any other GLP-1ra (Control Group). Patients were followed-up on for 24 months in the HF unit between January 2022 and December 2025. The diagnosis of HF was established according to the 2021 European Society of Cardiology Guidelines [17]. In accordance with these guidelines, patients with HFrEF were defined as individuals with signs and symptoms of HF, with evidence of structural and/or functional heart abnormalities and/or increased natriuretic peptides, and with a left ventricular ejection fraction (LVEF) ≤ 40%. Obesity was defined as a body mass index (BMI) greater than or equal to 30. Presence of type 2 DM was determined if there was a type 2 DM diagnosis according to the American Diabetes Association definition (“Standards of Medical Care in Diabetes-2022”) the medical record [37]. Baseline characteristics, covariates, and outcome measures were extracted from routinely collected data available in the electronic health records of our public healthcare system. Patients who did not provide written informed consent for the consultation of medical records were excluded.

Patients in the Oral-Sema Group started a once-daily 3 mg dose of semaglutide for four weeks that could be increased to 7 mg for the following four weeks until they reached the maintenance dose of 7 mg or 14 mg, according to the healthcare professionals’ clinical judgment. For patients who had previously received GLP-1ras, it was possible to start with a 7 mg dose of semaglutide. During follow-up, all patients received general recommendations on a healthy diet and physical activity according to their functional class. Treatments with lipid-lowering drugs, antihypertensive agents, and diuretics were modified, if necessary, as per the healthcare professionals’ judgment.

Follow-up was conducted every three to four months and data on a multitude of clinical variables were collected at each evaluation following routine clinical care in the HF unit. Anthropometric data (body weight, BMI, and waist circumference), sociodemographic variables, clinical variables (type 2 DM duration and treatment, principal cause of HF, HF duration, LVEF, previous medical history and medication, New York Heart Association (NYHA) classification, total symptom score on the Spanish version of the Kansas City Cardiomyopathy Questionnaire (KCCQ) [38]), and laboratory variables (serum creatinine, estimated glomerular filtration rate measured using the Chronic Kidney Disease Epidemiology Collaboration (CKD-EPI) equation [39], basal fasting blood glucose (BG), glycated hemoglobin (HbA1c), LDL cholesterol, HDL cholesterol, total cholesterol, triglycerides, uric acid, hematocrit, N-terminal pro-brain natriuretic peptide (NT-pro-BNP), and urinary albumin/creatinine ratio) were collected at each evaluation. Adverse drug reactions, the need to discontinue semaglutide due to adverse events, cardiovascular events, HF events (emergency department visit due to HF decompensation, hospitalizations due to HF, and unplanned outpatient visits), all-cause hospitalizations, and death (all-cause and cardiovascular) were also recorded.

The study was approved by the Institutional Research Ethics Committee of Málaga (Code: REDI-v4-26—18 July 2022) and written informed consent for the consultation of medical records was obtained from all participants. This study was conducted in accordance with the Declaration of Helsinki. Data confidentiality and patient anonymity were maintained at all times.

### 4.2. Outcome Measures

The primary outcome measure was HF health status, defined as a difference ≥5 points on the Spanish version of the KCCQ total symptom score, and change in body weight at 24 months. Secondary outcomes included the number of HF events (defined as a composite of emergency department visits due to HF decompensation, HF hospitalizations, and unplanned outpatient visits), cardiovascular death, all-cause death, all-cause hospitalizations, and new or worsening nephropathy (persistent macroalbuminuria, persistent doubling of the serum creatinine level and a creatinine clearance of <45 mL/min/1.73 m^2^, or the need for continuous renal-replacement therapy). Glycemic efficacy, as determined by the reduction in HbA1c, was also evaluated.

### 4.3. Statistical Analyses

The characteristics of patients included in this study were analyzed using descriptive statistics. Continuous and categorical variables were expressed as means ± standard deviation and as absolute values and percentages, respectively. The differences between groups were determined using the two-sample Student’s *t*-test or the Mann–Whitney–Wilcoxon rank-sum test for continuous variables and Pearson’s chi-square for categorical variables. The Pearson correlation coefficient was calculated to estimate the linear correlations.

Patients were grouped according to use of once-daily oral semaglutide. In order to match each patient in each group, a 1:1 propensity score matching (PSM) with a caliper of 0.2 and a greedy matching algorithm were used. Covariate balance before and after PSM was additionally assessed using standardized mean difference plots (Love plots). PSM adequacy was assessed using the standardized difference (a standardized difference >10% between baseline variables was defined as a significant imbalance). The probability of starting semaglutide was estimated using a logistic regression model that included variables that could have affected treatment assignment or outcomes as independent variables (sex, age, anthropometric characteristics, previous medical history, type 2 DM and HF characteristics, and laboratory findings). In order to evaluate the association between treatment and HF health status and secondary outcomes, mixed effect logistic regressions were used and adjusted for confounding variables (sex, age, anthropometric characteristics, previous medical history, type 2 DM and HF characteristics, and laboratory findings). Given that the KCCQ total symptom score is a bounded outcome ranging from 0 to 100, sensitivity analyses were additionally performed using models accounting for baseline-dependent treatment effects, including an analysis of covariance interaction model between treatment assignment and baseline KCCQ total symptom score values, in order to ensure robustness of treatment effects across different baseline symptom severities. The change in body weight was evaluated using analysis of covariance, with the change at 24 months as the dependent variable. The regression analysis values were expressed as odds ratios (OR) and 95% confidence intervals (95% CI). Deaths were treated as clinical outcomes, whereas loss to follow-up was defined as failure to complete follow-up for reasons other than death. Missing data were handled using available-case analyses because the proportion of missing values was low and was not considered sufficient to materially influence the results. No formal adjustment for multiplicity was performed, as the primary outcome was prespecified and analyses of secondary outcomes were considered exploratory. Statistical significance was defined as *p* < 0.05. Statistical analyses were performed using SPSS Statistics for Windows, version 22.0 (IBM SPSS Statistics for Windows, IBM Corporation, Armonk, NY, USA).

## 5. Conclusions

In this real-world observational cohort, once-daily oral semaglutide was associated with improvements in HF health status and body weight reduction in patients with HFrEF, type 2 DM, and obesity. In addition, there were reductions in the HF events, cardiovascular death, and all-cause hospitalizations. Its tolerability and safety were good, with only a few gastrointestinal adverse events. These findings should be considered hypothesis-generating and warrant confirmation in adequately powered randomized controlled trials.

## Figures and Tables

**Table 1:** Baseline sociodemographic, clinical, and therapeutic characteristics of patients before and after the propensity score matching analysis.

	Pre-Propensity Score Matching Analysis				Post-Propensity Score Matching Analysis			
Variables	Oral-Sema Group (*n* = 231)	Control Group (*n* = 216)	Standardized Difference	*p*-Value	Oral-Sema Group (*n* = 162)	Control Group (*n* = 162)	Standardized Difference	*p*-Value
Sociodemographic characteristics								
Age (years)	71.5 ± 10.5	72.8 ± 11.2	0.058	0.187	72.0 ± 10.8	72.5 ± 11.0	0.081	0.190
Male	117 (50.6%)	112 (51.9%)	0.049	0.199	82 (50.6%)	84 (51.9%)	0.045	0.201
Anthropometric characteristics								
Body weight (kg)	92.0 ± 15.2	90.1 ± 14.0	0.048	0.199	91.2 ± 15.0	90.5 ± 14.2	0.041	0.204
Body Mass Index (kg/m^2^)	32.8 ± 2.5	31.5 ± 1.5	0.050	0.197	32.0 ± 2.0	31.8 ± 1.5	0.017	0.211
Obesity (Body Mass Index ≥ 30)	231 (100.0%)	216 (100.0%)	0.004	0.289	162 (100.0%)	162 (100.0%)	0.003	0.291
Waist circumference (cm)	127.2 ± 17.2	124.4 ± 15.5	0.055	0.188	126.5 ± 17.0	125.4 ± 16.2	0.042	0.202
SBP (mmHg)	124.5 ± 11.5	120.0 ± 10.0	0.061	0.176	124.0 ± 11.0	122.5 ± 10.5	0.051	0.182
DBP (mmHg)	71.0 ± 9.0	70.0 ± 8.5	0.015	0.213	71.0 ± 9.0	70.5 ± 8.8	0.011	0.219
Heart rate (bpm)	69.0 ± 10.0	68.0 ± 9.0	0.020	0.201	69.0 ± 10.0	68.5 ± 9.5	0.015	0.211
Diabetes characteristics								
Diabetes duration (years)	12.8 ± 7.5	11.2 ± 6.5	0.065	0.165	12.0 ± 7.1	11.8 ± 6.9	0.042	0.201
Patients with HbA1c < 7%	17 (7.4%)	51 (23.6%)	0.122	0.018	16 (9.9%)	29 (17.9%)	0.086	0.072
Diabetes therapy			0.079	0.101			0.068	0.129
Metformin	127 (55.0%)	129 (59.7%)			92 (56.8%)	95 (58.6%)		
Sulfonylurea	4 (1.7%)	5 (2.3%)			3 (1.9%)	3 (1.9%)		
DPP-4 inhibitor	90 (39.0%)	98 (45.4%)			65 (40.1%)	68 (42.0%)		
GLP-1 receptor agonist (not semaglutide)	46 (19.9%)	0			29 (17.9%)	0		
SGLT-2 inhibitor	138 (59.7%)	131 (60.6%)			97 (59.9%)	98 (60.5%)		
Basal insulin	69 (30.0%)	54 (25.0%)			45 (27.8%)	41 (25.3%)		
Basal insulin dose (Units/day)	15.0 ± 10.5	14.0 ± 10.0			14.0 ± 10.2	14.0 ± 10.0		
Insulin combinations	12 (5.2%)	10 (4.6%)			8 (4.9%)	8 (4.9%)		
Statins	215 (93.1%)	196 (90.7%)	0.031	0.198	149 (92.0%)	147 (90.7%)	0.014	0.251
Heart failure characteristics								
Heart failure duration (years)	7.5 ± 3.5	7.8 ± 3.7	0.028	0.208	7.5 ± 3.5	7.7 ± 3.6	0.019	0.209
Principal cause of heart failure			0.051	0.151			0.026	0.215
Ischemic	189 (81.8%)	165 (76.4%)			129 (79.6%)	126 (77.8%)		
Non-ischemic	42 (18.2%)	51 (23.6%)			33 (20.4%)	36 (22.2%)		
KCCQ total symptom score	62.8 ± 22.2	64.5 ± 24.5	0.029	0.206	63.5 ± 23.5	64.0 ± 24.4	0.015	0.239
NYHA functional class			0.049	0.161			0.011	0.241
I	0	0			0	0		
II	161 (69.7%)	141 (65.3%)			109 (67.3%)	108 (66.6%)		
III	70 (30.3%)	75 (34.7%)			53 (32.7%)	54 (33.4%)		
IV	0	0			0	0		
Left ventricular ejection fraction (%)	36.0 ± 7.0	38.5 ± 8.0	0.028	0.207	37.5 ± 7.5	38.0 ± 7.8	0.020	0.221
Heart failure medication			0.062	0.134			0.048	0.159
Diuretic	184 (79.7%)	177 (81.9%)			130 (80.2%)	131 (80.9%)		
ACE inhibitor	22 (9.5%)	22 (10.2%)			16 (10.0%)	16 (10.0%)		
ARB	70 (30.3%)	67 (31.0%)			50 (30.9%)	50 (30.9%)		
Sacubitril-valsartan	116 (50.2%)	111 (51.4%)			82 (50.6%)	83 (51.2%)		
Beta-blocker	196 (84.8%)	185 (85.6%)			138 (85.2%)	138 (85.2%)		
Ivabradine	70 (30.3%)	60 (27.8%)			49 (30.2%)	47 (29.0%)		
Mineralocorticoid receptor antagonist	120 (51.9%)	118 (54.6%)			85 (52.5%)	87 (53.7%)		
Digitalis	31 (13.4%)	32 (14.8%)			22 (13.6%)	23 (14.2%)		
Anticoagulant	108 (46.8%)	95 (44.0%)			74 (45.7%)	72 (44.4%)		
Previous medical history								
History of smoking	137 (59.3%)	119 (55.1%)	0.059	0.138	93 (57.4%)	90 (55.5%)	0.042	0.155
History of alcohol abuse	24 (10.4%)	21 (9.7%)	0.039	0.189	16 (9.9%)	16 (9.9%)	0.017	0.239
Hypertension	219 (95.0%)	198 (91.7%)	0.047	0.163	152 (93.8%)	150 (92.6%)	0.021	0.235
Dyslipidemia	210 (90.9%)	190 (88.0%)	0.038	0.199	145 (89.5%)	144 (88.8%)	0.020	0.241
Chronic kidney disease stage ≥3	130 (56.3%)	129 (59.7%)	0.055	0.151	93 (57.4%)	95 (58.6%)	0.040	0.156
Cerebrovascular disease	32 (13.9%)	25 (11.6%)	0.052	0.156	21 (13.0%)	19 (11.7%)	0.045	0.161
Chronic obstructive pulmonary disease	63 (27.3%)	51 (23.6%)	0.043	0.169	42 (25.9%)	40 (24.6%)	0.030	0.172
Atrial fibrillation	104 (45.0%)	93 (43.1%)	0.041	0.175	72 (44.4%)	71 (43.8%)	0.024	0.138
Laboratory variables								
Glucose (mg/dL)	151.1 ± 38.2	141.05 ± 35.2	0.075	0.105	145.5 ± 37.3	143.2 ± 36.5	0.042	0.153
HbA1c (%)	7.8 ± 1.3	7.1 ± 1.1	0.118	0.041	7.4 ± 1.2	7.2 ± 1.1	0.075	0.106
Creatinine (mg/dL)	1.2 ± 0.6	1.1 ± 0.6	0.026	0.141	1.1 ± 0.6	1.1 ± 0.6	0.013	0.202
EGFR (mL/min/1.73 m^2^)	53.1 ± 20.2	56.8 ± 22.5	0.039	0.162	54.0 ± 21.2	55.5 ± 22.0	0.034	0.188
Uric acid (mg/dL)	6.5 ± 4.5	6.1 ± 4.2	0.041	0.160	6.3 ± 4.4	6.2 ± 4.3	0.029	0.157
Hematocrit (%)	42.9 ± 6.5	41.2 ± 5.5	0.035	0.170	42.5 ± 6.1	42.0 ± 5.7	0.018	0.194
NT-proBNP (pg/mL)	1279.5 ± 680.5	1110.0 ± 619.5	0.047	0.155	1210.5 ± 655.2	1125.5 ± 629.0	0.031	0.144
LDL cholesterol (mg/dL)	64.0 ± 20.5	70.0 ± 23.4	0.062	0.142	66.9 ± 21.0	69.0 ± 23.0	0.029	0.159
HDL cholesterol (mg/dL)	44.5 ± 9.5	39.9 ± 9.0	0.029	0.171	42.5 ± 9.2	40.7 ± 9.3	0.017	0.199
Total cholesterol (mg/dL)	145.7 ± 36.5	152.5 ± 40.9	0.065	0.139	148.5 ± 37.5	151.3 ± 40.0	0.044	0.151
Triglycerides (mg/dL)	143.0 ± 41.0	155.0 ± 50.1	0.069	0.128	149.5 ± 48.2	153.0 ± 50.0	0.040	0.162
Urinary albumin/creatinine ratio (mg/g)	53.0 ± 42.5	44.5 ± 40.0	0.072	0.130	50.5 ± 41.5	47.1 ± 40.8	0.072	0.109

Continuous data are shown as means (standard deviations) and qualitative data as absolute value and percentage. The differences between groups were determined using the two-sample Student’s *t*-test or the Mann–Whitney–Wilcoxon rank-sum test for continuous variables and Pearson’s chi-square for categorical variables. ACE: angiotensin-converting enzyme; ARB: angiotensin receptor blocker; DBP: diastolic blood pressure; DPP-4: dipeptidyl peptidase-4; EGFR: estimated glomerular filtration rate; GLP-1: glucagon-like peptide-1; HbA1c: glycated hemoglobin; KCCQ: Kansas City Cardiomyopathy Questionnaire; NT-proBNP: N-terminal pro-brain natriuretic peptide; NYHA: New York Heart Association; SBP: systolic blood pressure; SGLT-2: sodium−glucose cotransporter 2.

**Table 2:** Heart failure health status and secondary outcomes.

Outcomes	Oral-Sema Group (*n* = 162)	Control Group (*n* = 162)	OR (95% CI)	*p*-Value
HF health status (*n*, %)	72 (44.4)	23 (14.2)	2.45 (1.25–3.65)	0.012
HF events (*n*, %)	35 (21.6)	64 (39.5)	0.84 (0.70–0.98)	0.018
Emergency visits for HF decompensation	31 (19.1)	48 (29.6)	0.86 (0.73–0.99)	0.024
HF hospitalization	20 (12.3)	49 (30.2)	0.84 (0.69–0.99)	0.020
Unplanned outpatient visits	33 (20.4)	56 (34.6)	0.87 (0.75–0.99)	0.039
Cardiovascular death (*n*, %)	16 (9.9)	33 (20.4)	0.88 (0.77–0.99)	0.035
All-cause death (*n*, %)	25 (15.4)	40 (24.7)	0.98 (0.78–1.18)	0.109
All-cause hospitalizations (*n*, %)	32 (19.8)	52 (32.1)	0.87 (0.76–0.98)	0.038
New or worsening nephropathy (*n*, %)	7 (4.3)	14 (8.6)	0.91 (0.70–1.12)	0.086

Data are shown as absolute values and percentages. In order to evaluate the association between treatment and study outcomes, mixed effect logistic regressions were used. The regression analysis values were expressed as odds ratio and 95% confidence interval. Values were considered to be statistically significant if *p* < 0.05. 95% CI: 95% confidence interval; HF: heart failure; OR: odds ratio.

## Data Availability

The original contributions presented in this study are included in the article. Further inquiries can be directed to the corresponding authors.
